# The current status of clinical trials focusing on nasopharyngeal carcinoma: A comprehensive analysis of ClinicalTrials.gov database

**DOI:** 10.1371/journal.pone.0196730

**Published:** 2018-05-02

**Authors:** Hao Peng, Lei Chen, Yu-Pei Chen, Wen-Fei Li, Ling-Long Tang, Ai-Hua Lin, Ying Sun, Jun Ma

**Affiliations:** 1 Department of Radiation Oncology, Sun Yat-sen University Cancer Center, State Key Laboratory of Oncology in Southern China, Collaborative Innovation Center for Cancer Medicine, Guangdong Key Laboratory of Nasopharyngeal Carcinoma Diagnosis and Therapy, Guangzhou, People’s Republic of China; 2 Department of Medical Statistics and Epidemiology, School of Public Health, Sun Yat-sen University, Guangzhou, People’s Republic of China; University of North Carolina at Chapel Hill, UNITED STATES

## Abstract

**Purpose:**

Clinical Trials have emerged as the main force in driving the development of medicine. However, little is known about the current status of clinical trials regarding nasopharyngeal carcinoma (NPC). This study aimed at providing a comprehensive landscape of NPC-related trials on the basis of ClinicalTrials.gov database.

**Patients and methods:**

We used the keyword “nasopharyngeal carcinoma” to search the ClinicalTrials.gov database and assessed the characteristics of these trials.

**Results:**

Up to December 30, 2016, 462 eligible trials in total were identified, of which 222 (48.0%) recruited only NPC (NPC trials) and the other 240 (52.0%) recruited both NPC and other cancers (multiple cancer trials). Moreover, 47 (10.2%) were Epstein-Barr virus (EBV)-related trials and 267 (57.8%) focused on metastatic/recurrent disease. Compared with NPC trials, the multiple cancer trials had a higher percentage of phase 1 (26.7% vs. 6.7%, *P* < 0.001) studies and more patients with metastatic/recurrent disease (72.5% vs. 41.9%, *P* < 0.001). Notably, non-EBV trials had more phase 2 or 3 (78.4% vs. 48.8%, *P* < 0.001) and interventional studies (89.5% vs. 70.7%, *P* = 0.002) than EBV trials. Obviously, more phase 2/3 or 3 trials were conducted in patients with non-metastatic/recurrent disease (29.4% vs. 4.9%, *P* < 0.001); however, metastatic/recurrent trials were more likely to be anticancer (94.6% vs. 63.6%, *P* < 0.001).

**Conclusions:**

The role of plasma EBV DNA in clinical trials is underestimated, and high-level randomized clinical trials should be performed for patients with metastatic/recurrent disease.

## Introduction

Nasopharyngeal carcinoma (NPC) differs from other head and neck cancers for its epidemiology, clinical characteristics and therapy modality; it has an incidence rate of 20 per 100,000 persons in endemic regions such as South East Asia and Southern China [1], and radiotherapy has come as the only curative treatment as a result of the anatomic constraints and its sensitivity to irradiation. With the advancement of radiotherapy technique and combined therapy strategies of radiotherapy and chemotherapy over the last twenty years, outcomes for NPC have improved greatly, producing a 5-year overall survival rate of 84.7–87.4% [2–4]. However, control of advanced disease may be unsatisfactory, with an overall survival of 67–77% [5]. Furthermore, distant metastasis at initial diagnosis or after radical radiotherapy and recurrent NPC still remain the most serious challenges as the median overall survival of these patients is only 20 months [6]. Therefore, much effort are urgently needed to develop more effective treatment modalities.

Clinical trials have emerging as foundation of evidence-based medicine and the main force in driving the development of medicine. In September 2004, a consensus has been reached by the International Committee of Medical Journal Editors (ICMJE) that clinical trials should be registered in a public registry before recruiting patients to ensure transparency of the whole process. Later on, this policy was applied to all the clinical trials starting recruitment after July 1, 2005 [7]. ClinicalTrials.gov, developed and maintained by National Library of Medicine (NLM), is a registry and results database of publicly and privately supported clinical studies of human participants conducted around the world. Currently, the ClinicalTrials.gov provides the most comprehensive source of information on ongoing and completed clinical studies worldwide.

As clinical trials usually represent the latest treatment modalities in the war against cancer, clinicians hope that these new drugs or technologies could be applied in clinical practice as soon as possible. Given the truth that we still lack a thorough understanding of current clinical studies regarding NPC, we therefore conducted this study aiming at providing a comprehensive landscape of NPC-related trials on the basis of ClinicalTrials.gov database and evaluating the characteristics of these studies.

## Materials and methods

### Data source and eligible study

**Fig 1:**
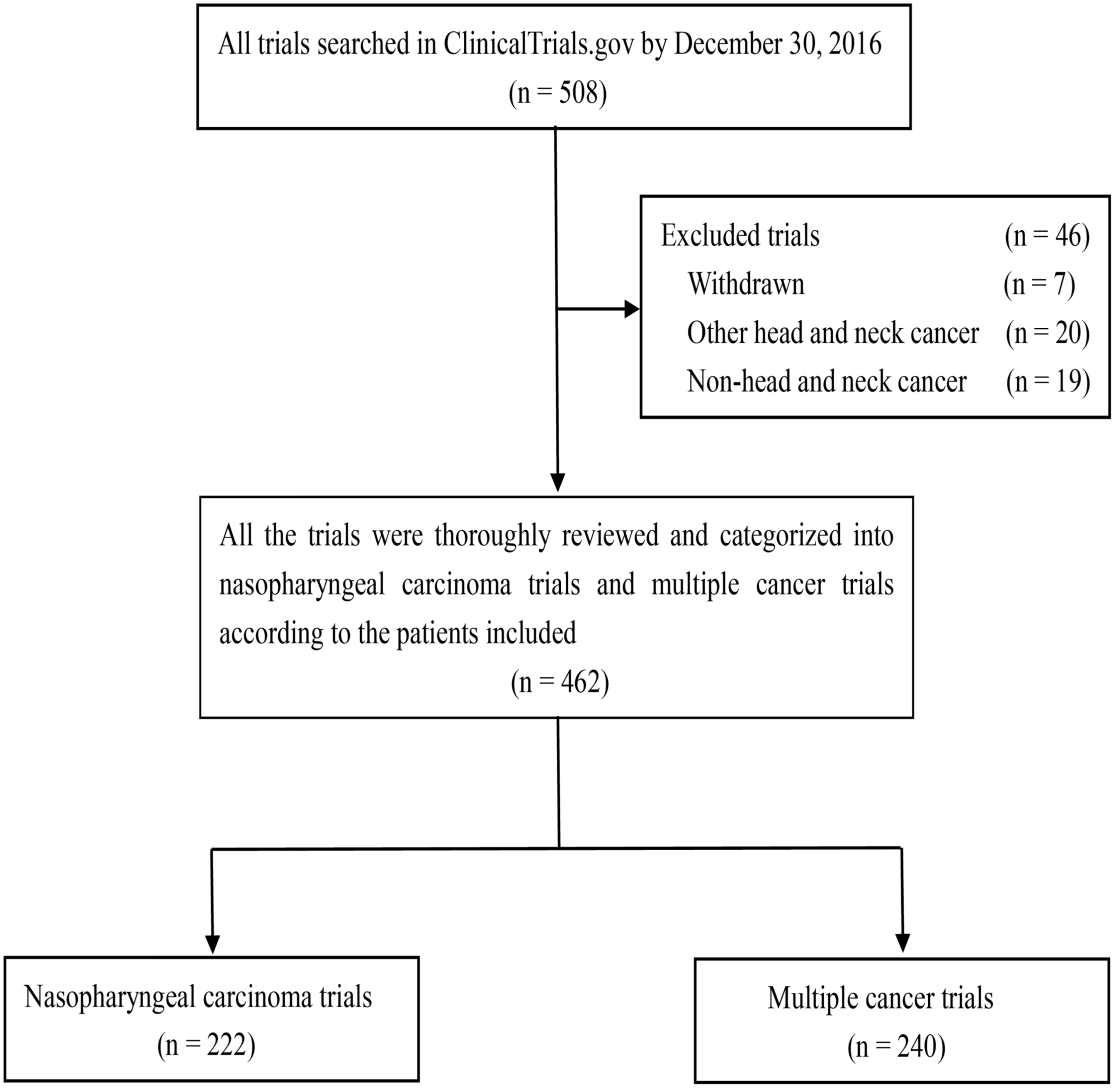
Flowchart of recruited NPC and multiple cancer trials registered with ClinicalTrials.gov by December 30, 2016. Abbreviations: NPC = nasopharyngeal carcinoma.

Three oncologists (LC, YPC and WFL) at the Sun Yat-sen University Cancer Centre used the term “nasopharyngeal carcinoma” to search all the registered clinical trials in the ClinicalTrials.gov database separately. All the information of these searched clinical trials provided by the sponsors and/or collaborators were thoroughly gone through and kept. A fourth oncologist (HP) would review the data recorded by the three oncologists, and any disagreements were solved by consensus or referring to the fifth oncologist (JM) who has more than twenty years of experience in NPC clinical trials. Up to December 30, 2016, a total of 508 trials were identified. After carefully reviewing all the information presented by ClinicalTrials.gov database, 46 (9.1%) trials were excluded (Fig 1).

Therefore, 462 (90.9%) trials were left for further analysis (S1 File). This study was approved by the Research Ethics Committee of Sun Yat-sen university cancer center.

### Study variables

Before searching, we set up recording standards for each study variable and the following characteristics provided by ClinicalTrials.gov database were assessed: registered number, registered time, Epstein-Barr virus (EBV)-related trials (yes or no), time perspective (prospective or retrospective), tumor stage (non-metastasis/recurrent or metastasis/recurrent or both or health population), tumor category (nasopharyngeal carcinoma only or multiple), the phase of trial (none or phase 0/1 or phase 1/2 or phase 2/3 or phase 4), study type (interventional or observational), interventional phase (none or prior to radiotherapy or during radiotherapy or after radiotherapy or metastatic/recurrent disease), interventional measure (none or anticancer or non-anticancer), anticancer drug (none or chemotherapy or targeted therapy or radiotherapy or immunotherapy or other), endpoint classification (efficacy or safety or efficacy/safety or other), masking (none or open label or blind), allocation (none or randomized or non-randomized), study arm (none or one or two or more), funding source (industry or national cancer institute or other), study sample (< 50 or 50–100 or > 100), participant age (< 18y or ≥ 18y or both), region (Unite states/Canada or European or Asia or other) and center (one or two or more).

The definition of EBV-related trials was that pre-treatment plasma EBV DNA was one of the inclusion criteria or therapy targeted EBV-related antigens such as latent membrane protein 1 (LMP1). If a trial included both NPC and other kinds of cancer types, it would be grouped into a “multiple” category. With regard to interventional stage, the trial would be classified as “metastatic/recurrent disease” if only patients with recurrent/metastatic disease were recruited; otherwise, the trial was categorized according to the order of intervention and radiotherapy for newly diagnosed, non-disseminated disease. For retrospective or observational studies, the phase of trial, interventional stage, interventional measure, anticancer drug, masking, allocation and study arm were considered as “none”, and the endpoint classification was “other”. Funding sources were categorized as industry, national cancer institute (NCI) or other academic groups based on the sponsor or collaborators [8]. If an industry was listed as the sponsor or collaborators, the trial would be treated as funded by industry. When NCI was the lead sponsor or collaborators, the trial was considered as NCI-funded. Furthermore, the region of the trial mainly depends on the location of lead sponsor.

### Statistical analysis

The characteristics of clinical trials were summarized by descriptive statistics: continuous variables were characterized as median and interquartile ranges (IQR) and categorical variables were reported as frequencies and percentages. Pearson Chi-square test was used to compare the characteristics difference between different kinds of NPC-related trials, and Fisher’s exact test would also be applied if indicated. Any missing value would be excluded from analysis. All statistical tests were performed using STATA version 13.0 (Stata Corporation LP, College Station, TX, USA), and a two-sided *P* < 0.05 was considered statistically significant.

## Results

### Basic characteristics of included trials

**Fig 2:**
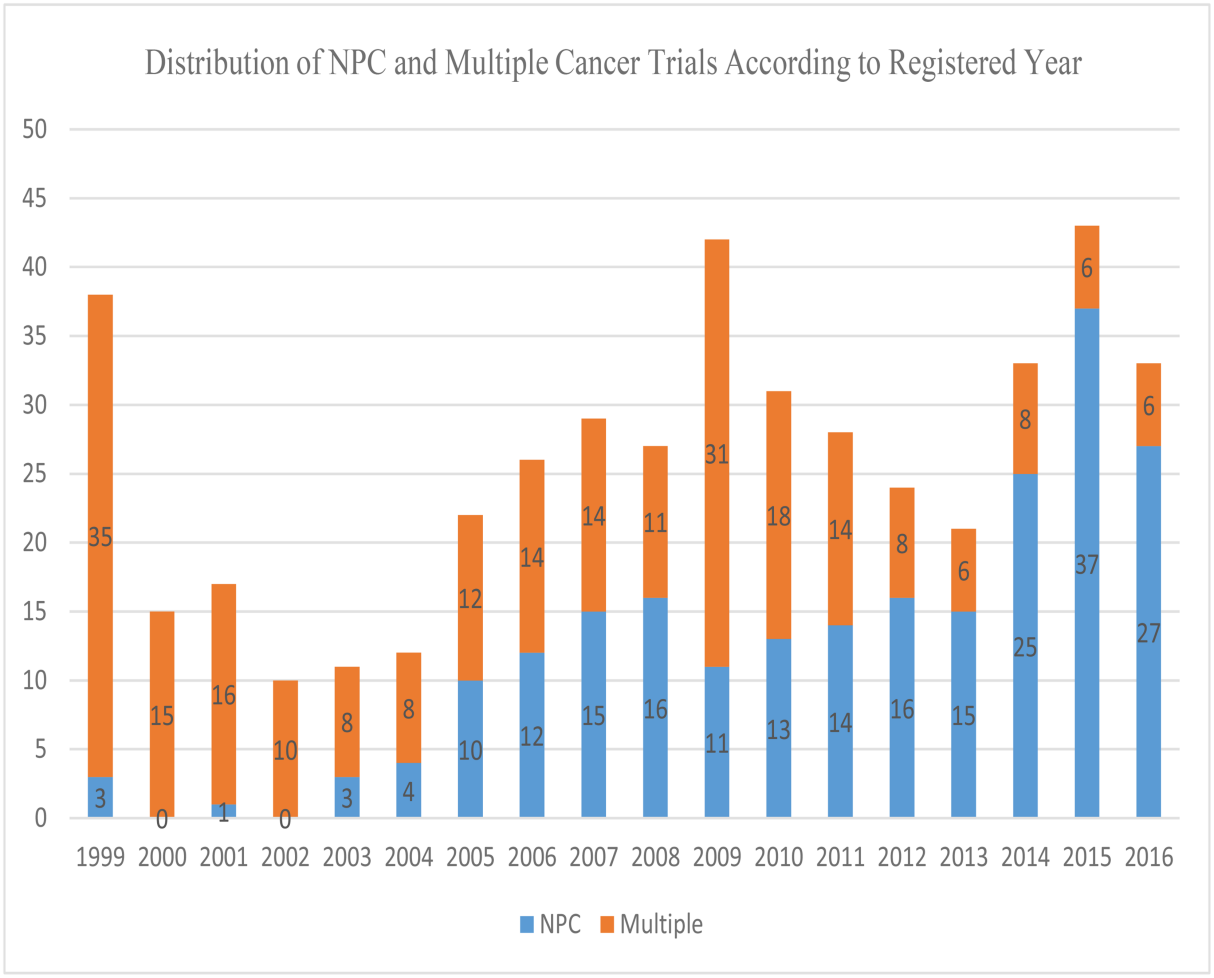
Distribution of NPC and multiple cancer trials according to registered year in ClinicalTrials.gov database. Abbreviations: NPC = nasopharyngeal carcinoma.

Among the 462 eligible trials, 222 (48.0%) were identified as NPC trials and the other 240 (52.0%) were multiple cancer trials. The distribution of these two kinds of trials according to registered time was summarized in Fig 2.

Obviously, the number of NPC trials increased greatly after 2004, and the number of multiple cancer trials decreased and remained stable after 2011. The baseline characteristics of 462 trials were presented in Table 1. Although plasma EBV DNA has been documented to be a reliable biomarker in prognosis predicting and decisions making in NPC since 2004 [9], its role in clinical trials still remains slight (10.2%). Intriguingly, more than half of trials (57.8%) focused on metastatic or recurrent disease and only 40.5% recruited non-disseminated NPC at initiation diagnosis. Notably, the primary purpose of most trials (72%) was anticancer intervention, and much attention was paid to chemotherapy (30.7%) and targeted therapy (23.4%). Moreover, 51.3% of the trials were registered in Unite States (US)/Canada where NPC has a very low rate of incidence, and most of these studies were multiple cancer trials which mainly focused on other head and neck cancers.

**Table 1:** Basic characteristics of the 462 trials registered with ClinicalTrials.gov up to December 30, 2016.

Characteristics	Number	Percentage (%)
**EBV-related trials**		
**Yes**	47	10.2
**No**	415	89.8
**Time perspective**		
**Prospective**	447	96.5
**Retrospective**	15	3.5
**Tumor stage**		
**Non-metastasis/recurrent**	187	40.5
**Metastasis/recurrent**	205	44.4
**Both**	62	13.4
**Health population**	8	1.7
**Tumor category**		
**NPC only**	222	48.0
**Multiple** ^a^	240	52.0
**Phase**		
**None**	77	16.7
**Phase 1**	81	17.5
**Phase 1/2 or 2**	203	43.9
**Phase 2/3 or 3**	69	14.9
**Phase 4**	5	1.1
**Missing value**	27	5.9
**Study type**		
**Interventional**	386	83.5
**Observational**	76	16.5
**Interventional phase**		
**None**	76	16.5
**Prior to radiotherapy**	37	8.0
**During radiotherapy**	93	20.1
**After radiotherapy**	33	7.1
**Metastatic/recurrent disease**	195	42.2
**Two or more phases**	25	5.4
**Missing value**	3	0.7
**Interventional measure**		
**None**	76	16.5
**Anticancer**	333	72.0
**Non-anticancer** ^b^	53	11.5
**Interventional drug**		
**None**	76	16.5
**Chemotherapy**	142	30.7
**Targeted therapy**	108	23.4
**Radiotherapy**	23	5.0
**Immunotherapy**	44	9.5
**Other** ^c^	69	14.9
**Endpoint classification**		
**Efficacy**	93	20.1
**Safety**	38	8.2
**Efficacy/safety**	254	55.0
**Other** ^d^	77	16.7
**Masking**		
**None**	77	16.7
**Open label**	348	75.3
**Blind**	37	8.0
**Allocation**		
**None**	77	16.7
**Randomized**	152	32.9
**Non-randomized**	233	50.4
**Study arm**		
**None**	77	16.7
**One**	229	49.6
**Two**	139	30.0
**Three or more**	17	3.7
**Funding source**		
**Industry**	46	10.0
**NCI**	174	37.6
**Other**	242	52.4
**Study sample**		
**< 50**	212	45.9
**50~100**	104	22.5
**> 100**	144	31.2
**Missing value**	2	0.4
**Participant age (y)**		
**< 18**	1	0.2
**≥ 18**	418	90.5
**Both**	43	9.3
**Region**		
**US/Canada**	237	51.3
**European**	26	5.6
**Asia**	195	42.2
**Other** ^e^	4	0.9
**Centers**		
**One**	299	64.7
**Two**	26	5.6
**Three or more**	137	29.7

Abbreviations: EBV = Epstein-Barr virus; NPC = nasopharyngeal carcinoma; NCI = national cancer institute; US = Unite States.

^a^ Trials includes both nasopharyngeal carcinoma and other kinds of cancer types.

^b^ Non-anticancer measures mainly include symptomatic treatment such as radiotherapy-induced oral mucositis.

^c^ Other refers to surgical treatment or drugs dealing with chemotherapy or radiotherapy-related toxicities.

^d^ Endpoint classifications of retrospective or prospectively observational study were considered as “other”.

^e^ Other regions include Africa, South America, Oceania, North America other than US/Canada.

### NPC trials and multiple cancer trials

Table 2 summarized the study characteristics of NPC trials and multiple cancer trials registered with ClinicalTrials.gov database. Difference in the number of EBV-related trials was apparent: NPC trials had an obviously higher rate of EBV-related trials (18.5% vs. 2.5%, *P* < 0.001) compared with multiple cancer trials. Moreover, NPC trials were more likely to focus on non-metastatic/recurrent disease at initiation diagnosis (55.0% vs. 27.1%, *P* < 0.001), while multiple cancer trials mainly recruited patients with metastatic/recurrent disease (55.0% vs. 32.0%, *P* < 0.001) and had more phase I studies (26.7% vs. 7.6%, *P* < 0.001). Unlike multiple cancer trials, NPC trials had a higher percentage of chemotherapy intervention (39.2% vs. 22.9%, *P* = 0.001). Furthermore, NPC trials were more likely to be funded by other academic groups (82.8% vs. 24.2%, *P* < 0.001) and had more large-scale studies (41.9% vs. 21.2%, *P* < 0.001) compared to multiple cancer trials. Obviously, most of NPC trials were conducted in Asia and multiple cancer trials in US/Canada.

**Table 2:** Characteristics difference between different trials registered with ClinicalTrials.gov up to December 30, 2016.

	NPC Trials	Multiple Cancer Trials		EBV Trials	Non-EBV Trials	
	(n = 222)	(n = 240)		(n = 41)	(n = 181)	
Characteristics	No. (%)	No. (%)	*P*_1_ ^a^	No. (%)	No. (%)	*P*_2_ ^a^
**EBV-related trials**			< 0.001			-
**Yes**	41 (18.5)	6 (2.5)		-	-	
**No**	181 (81.5	234 (97.5)		-	-	
**Time perspective**			0.246			0.587
**Prospective**	217 (97.7)	230 (95.8)		41 (100)	176 (97.2)	
**Retrospective**	5 (2.3)	10 (4.2)		0 (0)	5 (2.8)	
**Tumor stage**			< 0.001			< 0.001
**Non-metastatic/recurrent**	122 (55.0)	65 (27.1)		17 (41.5)	105 (58.0)	
**Metastatic/recurrent**	73 (32.9)	132 (55.0)		13 (31.7)	60 (33.1)	
**Both**	20 (9.0)	42 (17.5)		4 (9.8)	16 (8.9)	
**Health population**	7 (3.1)	1 (0.4)		7 (17.0)	0 (0)	
**Phase** ^b^			< 0.001			< 0.001
**None**	32 (14.4)	45 (18.8)		12 (29.3)	20 (11.0)	
**Phase 1**	17 (7.6)	64 (26.7)		9 (21.9)	8 (4.4)	
**Phase 1/2 or 2**	108 (48.6)	95 (39.6)		12 (29.3)	96 (53.0)	
**Phase 2/3 or 3**	54 (24.3)	15 (6.3)		8 (19.5)	46 (25.4)	
**Phase 4**	3 (1.4)	2 (0.8)		0 (0)	3 (1.7)	
**Study type**			0.166			0.002
**Interventional**	191 (86.0)	195 (81.2)		29 (70.7)	162 (89.5)	
**Observational**	31 (14.0)	45 (18.8)		12 (29.3)	19 (10.5)	
**Interventional phase** ^c^			< 0.001			0.003
**None**	31 (14.0)	45 (18.8)		12 (29.3)	19 (10.5)	
**Prior to radiotherapy**	32 (14.4)	5 (2.0)		2 (4.9)	30 (16.6)	
**During radiotherapy**	50 (22.5)	43 (17.9)		5 (12.2)	45 (24.9)	
**After radiotherapy**	22 (9.9)	11 (4.6)		7 (17.1)	15 (8.3)	
**Metastatic/recurrent disease**	70 (31.5)	125 (52.1)		14 (34.1)	56 (30.9)	
**Two or more phases**	15 (6.8)	10 (4.2)		1 (2.4)	14 (7.7)	
**Interventional measure**			0.07			< 0.001
**None**	31 (14.0)	45 (18.8)		12 (29.3)	19 (10.5)	
**Anticancer**	171 (77.0)	162 (67.5)		29 (70.7)	142 (78.5)	
**Non-anticancer**	20 (9.0)	33 (13.7)		0 (0)	20 (11.0)	
**Interventional drug**			0.001			< 0.001
**None**	31 (14.0)	45 (18.8)		12 (29.3)	19 (10.5)	
**Chemotherapy**	87 (39.2)	55 (22.9)		10 (24.4)	77 (42.5)	
**Targeted therapy**	46 (20.7)	62 (25.8)		7 (17.0)	39 (21.5)	
**Radiotherapy**	13 (5.9)	10 (4.2)		0 (0)	13 (7.2)	
**Immunotherapy**	23 (10.3)	21 (8.7)		12 (29.3)	11 (6.1)	
**Other**	22 (9.9)	47 (19.6)		0 (0)	22 (12.2)	
**Endpoint classification**			< 0.001			0.018
**Efficacy**	35 (15.8)	58 (24.2)		5 (12.2)	30 (16.6)	
**Safety**	11 (5.0)	27 (11.2)		3 (7.3)	8 (4.4)	
**Efficacy/safety**	144 (64.8)	110 (45.8)		21 (51.2)	123 (68.0)	
**Other**	32 (14.4)	45 (18.8)		12 (29.3)	20 (11.0)	
**Masking**			0.337			0.02
**None**	32 (14.4)	45 (18.8)		12 (29.3)	20 (11.0)	
**Open label**	174 (78.4)	174 (72.5)		27 (65.8)	147 (81.3)	
**Blind**	16 (7.2)	21 (8.7)		2 (4.9)	14 (7.7)	
**Allocation**			< 0.001			0.008
**None**	32 (14.4)	45 (18.8)		12 (29.3)	20 (11.0)	
**Randomized**	105 (47.3)	47 (19.6)		14 (34.1)	91 (50.3)	
**Non-randomized**	85 (38.3)	148 (61.6)		15 (36.6)	70 (38.7)	
**Study arm**			< 0.001			0.019
**None**	32 (14.4)	45 (18.8)		12 (29.3)	20 (11.0)	
**One**	83 (37.4)	146 (60.8)		15 (36.6)	68 (37.6)	
**Two**	96 (43.2)	43 (17.9)		12 (29.3)	84 (46.4)	
**Three or more**	11 (5.0)	6 (2.5)		2 (4.8)	9 (5.0)	
**Funding source**			< 0.001			< 0.001
**Industry**	25 (11.3)	21 (8.7)		0 (0)	25 (13.8)	
**NCI**	13 (5.9)	161 (67.1)		6 (14.6)	7 (3.9)	
**Other**	184 (82.8)	58 (24.2)		35 (85.4)	149 (82.3)	
**Study sample** ^d^			< 0.001			0.01
**< 50**	72 (32.4)	140 (58.3)		18 (43.9)	54 (29.8)	
**50~100**	57 (25.7)	47 (19.6)		3 (7.3)	54 (29.8)	
**>100**	93 (41.9)	51 (21.2)		20 (48.8)	73 (40.4)	
**Region**			< 0.001			0.006
**US/Canada**	34 (15.3)	203 (84.6)		14 (34.1)	20 (11.0)	
**European**	10 (4.5)	16 (6.7)		1 (2.4)	9 (5.0)	
**Asia**	176 (79.3)	19 (7.9)		26 (63.5)	150 (82.9)	
**Other**	2 (0.9)	2 (0.8)		0 (0)	2 (1.1)	

Abbreviations: NPC = nasopharyngeal carcinoma; EBV = Epstein-Barr virus; NCI = national cancer institute; US = Unite States.

^a^ *P*-Values were calculated using Pearson Chi-Square test or Fisher’s exact test if indicated.

^b^ 8 trials in the NPC trials arm and 19 trials in the multiple cancer trials arm were missing; 8 trials in Non-EBV trials arm were missing.

^c^ 2 trials in the NPC trials arm and 1 trial in multiple cancer trials arm were missing; 2 trials in Non-EBV trials arm were missing.

^d^ 2 trials in multiple cancer trials arm were missing.

### EBV and non-EBV trials

Apparently, multiple cancer trials registered in Unite States/Canada mainly focused on other head and neck cancers and EBV was not an inclusion criteria, we therefore excluded these trials when analyzing the characteristic difference between EBV and non-EBV trials (Table 2). EBV trials were less likely to recruit non-metastatic/recurrent disease (41.5% vs. 58.0%, *P* < 0.001) and had a higher percentage of health participants (17.0% vs. 0, *P* < 0.001). Besides, non-EBV trials had more phase 2 or 3 (78.4% vs. 48.8%, *P* < 0.001) and interventional studies (89.5% vs. 70.7%, *P* = 0.002). Also, the intervention of non-EBV trials mainly focused on chemotherapy (42.5% vs. 24.4%, *P* < 0.001) while EBV trials had an obviously higher rate of immunotherapy intervention (29.3% vs. 6.1%, *P* < 0.001). Furthermore, non-EBV trials were more likely to receive funding from industry (13.8% vs. 0, *P* < 0.001) and registered in Asia (82.9% vs. 63.5%, *P* = 0.006).

### Metastatic/Recurrent and Non-metastatic/Recurrent trials

As prognosis of non-metastatic/recurrent nasopharyngeal carcinoma is much better than that of metastatic/recurrent disease, we therefore further compared the characteristics difference between trials recruiting non-metastatic/recurrent and metastatic/recurrent patients (Table 3). Obviously, more phase 2/3 or 3 trials were conducted in patients with non-metastatic/recurrent disease (29.4% vs. 4.9%, *P* < 0.001); however, metastatic/recurrent trials were more likely to be anticancer (94.6% vs. 63.6%, *P* < 0.001). Moreover, metastatic/recurrent trials had a higher percentage of targeted therapy (35.6% vs. 16.0%, *P* < 0.001) and immunotherapy (18.1% vs. 2.2%, *P* < 0.001) interventions compared with non-metastatic/recurrent trials. In addition, non-metastatic/recurrent trials intended to be funded by other academic groups (70.6% vs. 35.6%, *P* < 0.001), be conducted in Asia (58.3% vs. 29.8%, *P* < 0.001) and have large-scale samples of more than 100 (45.4% vs. 14.2%, *P* < 0.001).

**Table 3:** Characteristics of Metastatic/Recurrent and Non-metastatic/Recurrent trials registered with ClinicalTrials.gov up to December 30, 2016.

	Metastatic/recurrent Trials	Non-metastatic/recurrent Trials	
	(n = 205)	(n = 187)	
Characteristics	No. (%)	No. (%)	*P*^a^
**Time perspective**			0.032
**Prospective**	198 (96.6)	186 (99.5)	
**Retrospective**	7 (3.4)	1 (0.5)	
**Phase** ^b^			< 0.001
**None**	11 (5.4)	27 (14.4)	
**Phase 1**	59 (28.8)	12 (6.4)	
**Phase 1/2 or 2**	122 (59.5)	71 (38.0)	
**Phase 2/3 or 3**	10 (4.9)	55 (29.4)	
**Phase 4**	0 (0)	3 (1.6)	
**Study type**			0.001
**Interventional**	195 (95.1)	160 (85.6)	
**Observational**	10 (4.9)	27 (14.4)	
**Interventional measure**			< 0.001
**None**	10 (4.9)	27 (14.4)	
**Anticancer**	194 (94.6)	119 (63.6)	
**Non-anticancer**	1 (0.5)	41 (22.0)	
**Interventional drug**			< 0.001
**None**	10 (4.9)	27 (14.4)	
**Chemotherapy**	65 (31.7)	70 (37.4)	
**Targeted therapy**	73 (35.6)	30 (16.0)	
**Radiotherapy**	6 (2.9)	15 (8.0)	
**Immunotherapy**	37 (18.1)	4 (2.2)	
**Other**	14 (6.8)	41 (22.0)	
**Endpoint classification**			< 0.001
**Efficacy**	35 (17.1)	52 (27.8)	
**Safety**	24 (11.7)	5 (2.7)	
**Efficacy/safety**	136 (66.3)	102 (54.5)	
**Other**	10 (4.9)	28 (15.0)	
**Masking**			< 0.001
**None**	11 (5.4)	27 (14.4)	
**Open label**	189 (92.2)	130 (69.6)	
**Blind**	5 (2.4)	30 (16.0)	
**Allocation**			< 0.001
**None**	11 (5.4)	27 (14.4)	
**Randomized**	34 (16.6)	109 (58.3)	
**Non-randomized**	160 (78.0)	51 (27.3)	
**Funding source**			< 0.001
**Industry**	29 (14.2)	12 (6.4)	
**NCI**	103 (50.2)	43 (23.0)	
**Other**	73 (35.6)	132 (70.6)	
**Study sample**			< 0.001
**< 50**	129 (62.9)	59 (31.6)	
**50~100**	47 (22.9)	43 (23.0)	
**>100**	29 (14.2)	85 (45.4)	
**Region**			< 0.001
**US/Canada**	136 (66.3)	66 (35.3)	
**European**	7 (3.4)	9 (4.8)	
**Asia**	61 (29.8)	109 (58.3)	
**Other**	1 (0.5)	3 (1.6)	

Abbreviations: NCI = national cancer institute; US = Unite States.

^a^ *P*-values were calculated using Pearson Chi-square test or Fisher’s exact test if indicated.

^b^ Three trials in the metastatic/recurrent trials arm and 19 trials in non-metastatic/recurrent trials arm were missing.

## Discussion

Clinical trials have play an irreplaceable role in changing clinical practice and decision making in medicine, especially for well-designed randomized clinical trials. NPC, known as a cancer rising from nasopharynx epithelium, is mainly prevalent in Southeast Asia, the Middle East and North Africa [10–12]. Therefore, given the overall low incidence rate worldwide, NPC does not attract the attention of most researches and little is known about the current status of clinical trials regarding NPC. To the best of our knowledge, our study is the first one to report the landscape of NPC-related trials and assess the characteristics of these trials. Our findings suggested that NPC-related trials were predominantly early-phase trials with small samples less than 100 and mainly focused on chemotherapy and targeted therapy intervention. Surprisingly, metastatic/recurrent disease even occupied a greater part in these trials. Obviously, NPC trials were more likely to be performed in Asia while multiple cancer trials were mainly conducted in US/Canada.

Although multiple cancer trials recruited patients with NPC, the information of managing NPC they provided may be very limited because most of these trials were conducted in US/Canada where the incidence of NPC is extremely low and mainly focused on other head and neck cancers. Actually, there are few publications regarding NPC from this region. Notably, compared with NPC trials, the multiple cancer trials had a higher percentage of phase 1 (26.7% vs. 6.7%) studies and patients with metastatic/recurrent disease (72.5% vs. 41.9%). One reasonable explanation is that these trials were conducted to test new drugs or new treatment modalities in patients with metastatic/recurrent who failed standard therapy. Hence, these trials were more likely to have small samples of less than 50 (58.3% vs. 32.4%) and to be single arm (60.8% vs. 37.4%) and non-randomized (61.6% vs. 38.3%).

**Fig 3:**
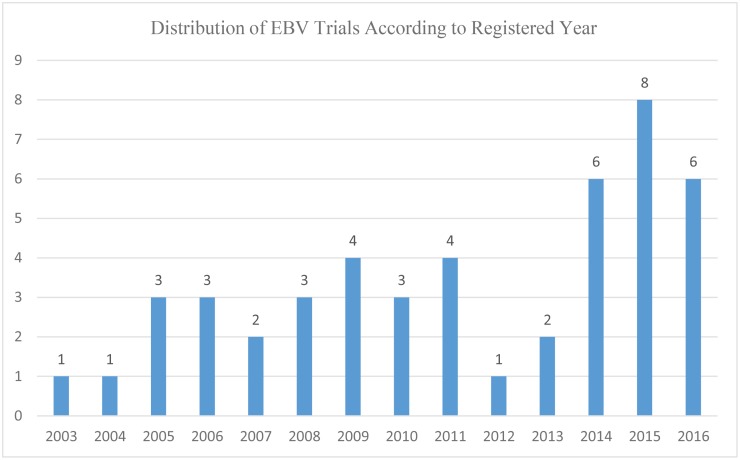
Distribution of EBV-related trials according to registered year in ClinicalTrials.gov database up. Abbreviations: EBV = Epstein-Barr virus.

NPC has been established as an EBV-associated cancer for a long time [13–15]. Subsequently, the prognostic value of plasma EBV DNA has been widely proven both in non-disseminated [9, 16–23] and metastatic/recurrent disease [24, 25]. Moreover, plasma EBV DNA could also stratify patients into different risk groups and guide individualized treatment [26–28]. Therefore, plasma EBV DNA could be a reliable biomarker and should play an important role when designing clinical trials. However, results of our study reveal only 10.2% of the trials are EBV-related, and the distribution of these trials (Fig 3) remind us that the number increased only after 2014 but was still small. One of the main reasons is that there is no uniform standard in detecting the plasma EBV DNA level worldwide and hospitals would get different results if different test reagents are used, which makes it hard to perform multicenter collaborations. Therefore, EBV trials has a lower percentage of phase 2/3 (48.8% vs. 78.4%) and interventional (70.7% vs. 89.5%) studies. Future trials are urgently warranted to focus on the standardization of detecting plasma EBV DNA.

Although primary metastasis at initial diagnosis accounts for only 4.4% to 6% of all NPC patients [29–31] and excellent therapeutic outcomes have been achieve for advanced NPC, distant metastasis and recurrence after radiotherapy still remain a huge challenge. Our study showed that 205 trials regarding metastatic/recurrent disease were performed; however, most of these trials were conducted in US/Canada and were multiple cancer trials mainly focusing on other head and neck cancers. Furthermore, these trials were more likely to be early-phase, non-randomized and small-scale (< 50) compared with trials recruiting non-metastatic/recurrent patients. Therefore, we still lack high-level evidence of managing metastatic/recurrent disease. Actually, a recent study carried out by Zhang et al. [32] is the only phase 3 randomized trial focusing on metastatic/recurrent disease in endemic era. Hence, more attention should be paid to this subpopulation to optimize clinical practice.

Limitations of this study should also be acknowledged. First, ClinicalTrials.gov database does not include all clinical trials because investigators and sponsors may register their studies at other registrations. This may be embedded in the small number of trials from European. Second, some investigators or sponsors may input unconsciously wrong information in this database which would complicate our conclusions as the NLM cannot verify the trial information sponsors provided on ClinicalTrials.gov. Moreover, we did not assess the final results of these trials because part of these trials are still ongoing or not reporting the results.

## Conclusions

Overall, our study firstly provides a best-possible overview of current clinical trials regarding NPC and demonstrated that the number is still insufficient especially for high-level, randomized phase 3 trials. The role of plasma EBV DNA in clinical trials is far from its value in clinical practice although numerous studies have established its value in prognosis prediction, risk stratification and decision making. Moreover, more randomized clinical trials should be performed for patients with metastatic/recurrent disease because we still lack high-level evidence in treating these patients.

## Supporting information

S1 File Summary of the 462 included clinical trials. (XLSX)
